# Comparison of LFP-Based and Spike-Based Spectro-Temporal Receptive Fields and Cross-Correlation in Cat Primary Auditory Cortex

**DOI:** 10.1371/journal.pone.0020046

**Published:** 2011-05-23

**Authors:** Jos J. Eggermont, Raymundo Munguia, Martin Pienkowski, Greg Shaw

**Affiliations:** 1 Department of Physiology and Pharmacology, University of Calgary, Calgary, Alberta, Canada; 2 Department of Psychology, University of Calgary, Calgary, Alberta, Canada; 3 Hotchkiss Brain Institute, University of Calgary, Calgary, Alberta, Canada; Claremont Colleges, United States of America

## Abstract

Multi-electrode array recordings of spike and local field potential (LFP) activity were made from primary auditory cortex of 12 normal hearing, ketamine-anesthetized cats. We evaluated 259 spectro-temporal receptive fields (STRFs) and 492 frequency-tuning curves (FTCs) based on LFPs and spikes simultaneously recorded on the same electrode. We compared their characteristic frequency (CF) gradients and their cross-correlation distances. The CF gradient for spike-based FTCs was about twice that for 2–40 Hz-filtered LFP-based FTCs, indicating greatly reduced frequency selectivity for LFPs. We also present comparisons for LFPs band-pass filtered between 4–8 Hz, 8–16 Hz and 16–40 Hz, with spike-based STRFs, on the basis of their marginal frequency distributions. We find on average a significantly larger correlation between the spike based marginal frequency distributions and those based on the 16–40 Hz filtered LFP, compared to those based on the 4–8 Hz, 8–16 Hz and 2–40 Hz filtered LFP. This suggests greater frequency specificity for the 16–40 Hz LFPs compared to those of lower frequency content. For spontaneous LFP and spike activity we evaluated 1373 pair correlations for pairs with >200 spikes in 900 s per electrode. Peak correlation-coefficient space constants were similar for the 2–40 Hz filtered LFP (5.5 mm) and the 16–40 Hz LFP (7.4 mm), whereas for spike-pair correlations it was about half that, at 3.2 mm. Comparing spike-pairs with 2–40 Hz (and 16–40 Hz) LFP-pair correlations showed that about 16% (9%) of the variance in the spike-pair correlations could be explained from LFP-pair correlations recorded on the same electrodes within the same electrode array. This larger correlation distance combined with the reduced CF gradient and much broader frequency selectivity suggests that LFPs are not a substitute for spike activity in primary auditory cortex.

## Introduction

Comparison of the receptive fields at the input and output of a cortical cell can reveal information of what the cell, under the influence of all its excitatory, inhibitory, and modulatory inputs, does. The output of a cortical cell is provided by its spiking activity. The cell's compound input of all presynaptic contributions is reflected in the postsynaptic potential (PSP). An extracellular reflection of many synchronous PSPs in a limited volume around the recording electrode is found in the short latency parts of the stimulus evoked local field potential (LFP).

Because the PSP results from the charging of membrane capacitors, the associated transmembrane current is proportional to the time derivative of the PSP. For synchronous activation of many cells in the recording volume of an extracellular electrode, one expects the LFP to be a weighted sum of the time derivative of these PSPs [1]. The LFP thus constitutes a collective property of a neuronal ensemble, i.e., is a measure of synchronous post-synaptic activity of a population of neurons. This ensemble activity may, besides excitatory PSPs (EPSPs), include inhibitory PSPs, subthreshold membrane potential oscillations, and afterpotentials of somatodendritic action potentials [2]. Currently, LFPs are used more and more in sensory [3] or in cognitive [4] prostheses. It is thus important to know how LFPs compare to multi-unit activity, especially with respect to their topographic specificity.

Frequency-tuning curves (FTCs) derived from EPSPs and LFPs from the same location in auditory cortex slice resembled each other in terms of characteristic frequency (CF) and frequency-tuning bandwidth, corroborating that LFPs reflect local synaptic (including subthreshold) activity from both thalamo-cortical and intracortical afferents [5]. Subthreshold EPSP- and LFP-based frequency-tuning curves however were remarkably broad, on average at least a factor 2 broader at 20 dB above threshold than those for spikes [5], [6]. This may be due to the convergence of many thalamic inputs with different CF onto the same cortical cell [7] or to horizontal fiber activity originating from other cortical cells with different CFs [8]. The narrower spike-based frequency-tuning curves appear to be the result of intra-cortical inhibition, since bicuculine application increases the width of the spike-based tuning curves to that of the LFPs [9]. However, the excitatory and inhibitory receptive fields of an auditory cortical neuron can cover almost the same frequency range (i.e., can be co-tuned), suggesting that inhibition sharpens spike-based tuning by simply reducing the amplitude (and bandwidth) of the depolarization that would be produced by excitation alone [10], [11]. On the other hand, a more recent study has shown that inhibitory inputs to primary auditory cortex (AI) neurons can in fact be more broadly tuned than excitatory ones, implying a sharpening of spike tuning by classical surround inhibition [12]. It has also been shown that the bandwidth of spike-based frequency-tuning curves for auditory thalamic and cortical neurons is nearly the same [7] and that the intra-cortical inhibition has to recreate this narrow bandwidth from the more broadly tuned input, as reflected in the LFP.

FTCs offer only a static picture of the neuron's spectral sensitivity. The temporal aspects of cortical frequency tuning can be studied with spectro-temporal receptive fields (STRF), depicting the frequency tuning as a function of time after a particular stimulus fragment embedded in segments with other frequencies [13], [14], [15]. It is clear that in auditory cortex STRFs based on sub-threshold membrane potentials and LFPs are spectrally broader and longer lasting than those based on spikes [16], [17].

Here, we compare FTCs and STRFs obtained from simultaneously recorded multiple sorted unit (MSU) spike and 2–40 Hz filtered LFP activity, using multi-electrode arrays with 8 or 16 electrodes. We find that LFPs in the 2–40 Hz frequency range in primary auditory cortex (AI), are much less frequency and place selective than sorted-spike activity. In addition we constructed STRFs based on 4–8 Hz, 8–16 Hz and 16–40 Hz filtered LFPs and demonstrate that the correlation with spike-based STRF estimates improves for the 16–40 Hz filtered LFPs.

In sleeping or ketamine-anesthetized cats there is a strong spatial correlation between LFP activity on electrodes with up to 7 mm separation in neocortex [18], i.e., about the extent of cat AI. However, spike-synchrony based neuron clusters are generally smaller and only up to 1 mm in size [19]. It would thus be of considerable interest to compare the space constants of correlated spike-pair activity and correlated LFP-pair activity recorded on the same set of electrodes. Here, we compare spatial correlations obtained from simultaneously recorded spontaneous MSU with 2–40 Hz and 16–40 Hz filtered LFP activity in AI. We show that the space constant of the cross-correlogram peaks is about a factor 2 larger for LFP-pair correlations compared to spike-pair correlations.

## Materials and Methods

### Ethics statement

The care and the use of animals reported in this study was approved (BI 2007-12) and reviewed on a yearly basis by the Life and Environmental Sciences Animal Care Committee of the University of Calgary. All animals were maintained and handled according to the guidelines set by the Canadian Council of Animal Care.

### Animal preparation

All animals were deeply anesthetized with the administration of 25 mg/kg of ketamine hydrochloride and 20 mg/kg of sodium pentobarbital, injected intramuscularly. A mixture of 0.2 ml of acepromazine (0.25 mg/ml) and 0.8 ml of atropine methyl nitrate (25 mg/ml) was administered subcutaneously at approximately 0.25 ml/kg body weight. Lidocaine (20 mg/ml) was injected subcutaneously prior to incision. The tissue overlying the right temporal lobe was removed and the dura was resected to expose the area bounded by anterior and posterior ectosylvian sulci. The cat was then secured with one screw cemented on the head without any other restraint. The wound margins were infused every 2 hours with lidocaine, and additional acepromazine/atropine mixture was administered every 2 hours. The ketamine dose to maintain a state of non-reflexive anesthesia was on average 12 (SD = 4) mg/kg*h.

### Acoustic stimulus presentation

Stimuli were generated in MATLAB® and transferred to the DSP boards of a TDT-2 (Tucker Davis Technologies) sound delivery system. Acoustic stimuli were presented in an anechoic room from a speaker system (Fostex RM765 in combination with a Realistic Super-Tweeter that produced a flat spectrum (±5 dB) up to 40 kHz measured at the cat's head) placed approximately 30 degrees from the midline into the contralateral field, about 50 cm from the cat's left ear. Calibration and monitoring of the sound field was accomplished with a condenser microphone (Bruel & Kjaer 4134) placed above the animal's head, facing the speaker and a measuring amplifier (Bruel & Kjaer 2636). Prior to acute recordings peripheral hearing sensitivity was determined using auditory brainstem response (ABR) thresholds [20].

### Frequency-tuning properties

Intensity-frequency-tuning curves were measured by randomly presenting 27 or 38 gamma-tone pips with frequencies covering 5 or 7 octaves (e.g., 1.25–40 kHz or 0.3–40 kHz) in equal logarithmic steps and presented at 8 different stimulus levels in 10 dB steps (i.e., 5–75 dB SPL) at a rate of 4/s such that each intensity-frequency combination was repeated 10 times. The envelope of the gamma tones is given by:
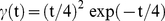
(1)with 0≤t≤50 ms. Thus, amplitudes exceeded half-max over ∼3–17 ms post pip onset, and were truncated at 50 ms where the amplitude is down by 64 dB.

Spectro-temporal receptive fields (STRF) were obtained by presenting multi-frequency stimuli consisting of randomly presented gamma-tone pips, equally spaced on a logarithmic scale. Here, tone pips for each of 81 frequencies in 5 octaves were randomly presented according to a Poisson process [21], with similar average rate but different realization for each frequency. The stimulus ensemble used had an aggregate tone pip rate of 20/s. In other words, the presentation of tone pips for each of the 81 frequencies was a realization of a Poisson process with a mean tone-pip rate of 0.25 Hz. For each of these frequencies the realization was different. By pooling these 81 different random tone sequences, where each tone-pip has a 50 ms duration with a half-width of 14 ms, there will be temporal overlaps of tone-pips with different frequencies. The presentation level was 65 dB SPL (peak-equivalent).

### Recording and spike separation procedure

Two arrays of 8 or 16 electrodes each (Microprobe Inc.), with impedances between 1 and 2 MΩ, were used for the simultaneous extracellular recording of spikes and local field potentials (LFPs) using high-impedance head stages (RA16AC, Tucker Davis Technologies). Electrodes were arranged in a 2×4 (2×8) configuration with a separation of 0.5 mm between the two rows and 0.5 (0.25) mm between adjacent columns. The reference/ground electrode was placed in the neck muscle. The arrays were oriented such that all electrodes were touching the cortical surface roughly orthogonally, and then each array was manually advanced using a hydraulic microdrive (Narishige M101). The depth of recording was between 700 and 1200 µm and thus the electrodes were likely in deep layer III or layer IV. Recorded potentials were amplified 10^4^ times using a pair of amplifiers (RA16PA, Tucker Davis Technologies) and processed by a multichannel data acquisition system (RX5, Tucker Davis Technologies). Spikes were identified online (using trigger levels set well outside the noise floor) from the 300 Hz-3 kHz filtered electrode signal, and LFPs were obtained from the 2–40 Hz filtered electrode signal. Narrow band LFPs were obtained by steep filtering with 8^th^ order (both sides) Butterworth filters at 4–8 Hz, 8–16 Hz and 16–40 Hz (−3 dB cut-off frequencies).

Filter delays in the LFP signal were corrected before obtaining the LFP averages. Spike sorting was done off-line using a semi-automated procedure based on principal component analysis and K-means clustering implemented in MATLAB. The spike times and waveforms were stored. The multiple sorted-unit (MSU) data presented in this paper represent only well sorted units that, because of their regular spike wave form, likely are dominantly from pyramidal cells. For statistical purposes, the sorted-unit spike trains were pooled to form a MSU spike train, thereby eliminating potential contributions from thalamocortical afferents or fast spikes from interneurons.

### Data analysis

#### FTC

To assess frequency-tuning properties, the peak number of action potentials in a 5 ms bin of the post-stimulus time histogram over the first 100 ms for each frequency presentation was estimated. The counts were divided by the number of stimuli and presented as a firing rate per stimulus. The results were calculated per stimulus intensity, and were combined into an intensity-frequency-rate profile from which tuning curves were derived using routines implemented in MATLAB. The FTC bandwidth was defined at 25% of the maximum peak-firing rate. This was about 10–20% above the spontaneous firing rate, but as the latter was dependent on the level of stimulus-induced suppression, the criterion based on peak firing rate was preferred. This bandwidth was measured at 20 dB above threshold at CF (BW_20 dB_) and expressed in octaves.

#### STRF

**Figure 1:**
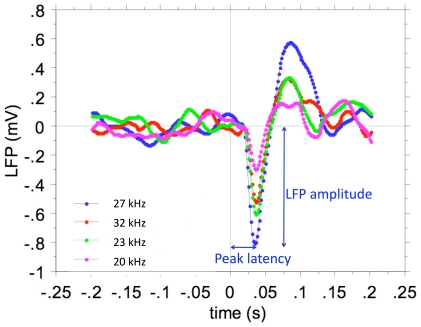
Stimulus-triggered average LFPs for different tone frequencies at and around the BF (27 kHz). Peak amplitude is measured from baseline and peak latency from tone pip onset.

Our methodology for computing STRFs from random chords with high-time resolution was developed by [15] and [21]. Briefly, STRFs for MSU were determined by constructing poststimulus time histograms (PSTHs), with time bins of 1 ms, for each frequency. In other words, spikes falling in the averaging time window (starting at the stimulus onset and lasting 100 ms) are counted. Because the average interstimulus interval in the stimulus ensemble is smaller (3.6 ms) than the averaging time window (100 ms), a spike can be counted in the PSTH of several pip frequencies. The mean firing rate per bin, obtained by dividing the number of spikes per second in a bin by the number of tone pips, is the dependent variable displayed in the MSU STRFs. We showed previously that STRFs calculated this way have higher temporal resolution than those based on reverse correlation, and identical frequency resolution [15]. STRFs for LFPs were obtained by a similar procedure, except that the stimulus-evoked LFP waveforms (0–100 ms after stimulus onset) were averaged for each appropriate tone pip frequency. Figure 1 shows an example of such stimulus triggered 2–40 Hz filtered LFPs for 4 different tone frequencies of the multi-frequency stimulus ensemble at and around the best frequency (BF). Peak amplitude is measured from baseline and peak latency from tone pip onset. The amplitude decreases with frequency distance from the BF, whereas the latency increases. For this unit in AI and tuned at BF = 27 kHz, the response at 20 kHz is only about 1/3^rd^ of the value at the BF, indicating relatively sharp tuning. For display purposes all STRFs were smoothed with a uniform 5×5 bin (1/3^rd^ oct.×5 ms) window.

For spike-based STRFs, all obtained at 65 dB SPL, color-coded images are plotted superimposed on the LFP-based STRFs shown as contour lines at 25, 50 and 75% of maximum.

Comparisons of STRFs for band-pass filtered LFP signals and spike-based STRFs were based on the marginal frequency distributions of the STRF RMS values because of the oscillatory character of the 4–8, 8–16 and 16–40 Hz band-passed LFP-based STRFs. The comparison was quantified by the correlation coefficients of the 81 frequency-point sequences for each of the distributions.

#### Cortical area boundaries

The following properties were used in the assessment of cortical area boundaries: reversal of the CF gradient in the tonotopic map and along the electrode array, minimum latency values, the shape of the STRF, and the peak value of the cross-correlation coefficient for recordings straddling boundaries. For delineating the border between primary auditory cortex (AI) and anterior auditory field (AAF), we first of all used the sign and/or reversal of the gradient of CF along the electrode array with distance in the anterior direction [22]. The general shorter minimum latency in AAF compared to AI, and particularly the much higher frequency-tuning curve bandwidth at 20 dB above threshold in AAF [23] were used as well. For the distinction between AI and posterior auditory field (PAF) or potentially EPI (intermediate part of the posterior ectosylvian gyrus) we used mainly latency, which was at least 20 ms larger in these non-AI areas. EPI was characterized by very fuzzy STRFs, especially for spikes. In addition, the sudden drop in peak cross-correlation coefficient across area boundaries under spontaneous firing conditions [24] was a highly consistent indicator. The dorsal zone of AI was identified based on its more complex frequency tuning, a different frequency gradient, and latencies in between those of AI and PAF, whereas secondary auditory cortex was characterized by its general lack of a tonotopic gradient. We only used data from AI in the present paper.

#### Cross-correlation functions

Recordings of spontaneous activity were obtained for contiguous periods of 15 minutes. Only stationary data were considered [25] Correlations between spikes recorded simultaneously on pairs of electrodes and between LFPs recorded on pairs of electrodes were investigated under spontaneous conditions, i.e., without sound stimulation. The cross-correlation between spikes and LFPs was done through a spike-triggered averaging (STA) of LFPs recorded on the same electrode as the MSU.

For pairs of LFP recordings, the cross-correlation function is first computed as [26]

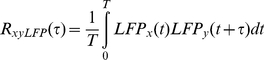
(2)which is then normalized by subtracting the product of the mean values μ_x_μ_y_ to obtain the cross-covariance C_xyLFP_(τ). The cross-correlation coefficient function is then obtained by dividing with the square root of the autocovariances, C_xxLFP_(τ) and C_yyLFP_(τ) taken at τ = 0.
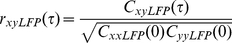
(3)The peak value of r_xyLFP_(τ) is indicated by r_xy_(LFP).

In addition we used the inverse Fourier transform (IFT) of the complex coherence function γ_xyLFP_(f) of the x and y recordings as an estimate of a cross-correlation coefficient rc_xyLFP_(τ) corrected for common periodicities such as delta or spindle oscillations.
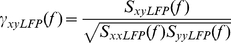
(4)

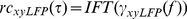
(5)The peak value of rcxyLFP(τ) is indicated by RCxy(LFP).

Spike-spike cross-correlograms were calculated as previously described [21], [27] using the cross-correlation coefficient function:
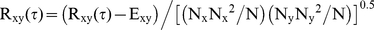
(6)where R_xy_(τ) is the number of coincidences in the bin corresponding to lag time τ, E_xy_ is the expected value for coincidences under the assumption of independent spike trains, E_xy_ = (N_x_N_y_)/N, with N = T/Δ, where N_x_ and N_y_ are the number of spikes in the recording, Δ is the bin size, and T the duration of the recording. |R(τ)|≤1. Stationarity estimates of the recordings were based on firing rate (mean and variance) in 100 second long segments of the 15-minute recordings.

Functional correlation strength is not independent of the firing properties of the neurons in the pair. Specifically, periodicities in the neuronal firings imposed by cortical oscillations, e.g., in the spindle frequency range, may affect the peak cross-correlation coefficient [28]. Thus a deconvolution of the cross-covariance by the geometric mean of the auto-covariance functions of the two spike trains was implemented here. To correct for the overall firing rate, burst firing and periodicities in the firing of the neurons, the cross covariance, (R_xy_(τ)−E_xy_), was deconvolved with the square root of the product of the autocovariance functions, (R_xx_(τ)−E_xx_) and (R_yy_(τ)−E_yy_). Here E_xy_ and E_xx_ and E_yy_ are the expected values for the cross- respectively auto-correlation functions under the assumption of independence and Poisson processes. This deconvolution was done in the frequency domain, where it becomes a simple division, and is similar to calculating the complex coherence function. Fourier transformation back to the time domain resulted in the coherence-corrected cross-correlation coefficient function RC_xy_(τ). The cross-correlations were all significantly different from zero at a level of 3 SD (p<0.01). The peak value of RC_xy_(τ) is indicated by RC_xy_(sp).

All statistical analyses were performed using Statview 5® (SAS Institute Inc.).

## Results

### Stimulus evoked activity

Multi-electrode array recordings were obtained from AI of 12 normal hearing cats. FTCs (n = 398) that allowed unambiguous estimation of CF, bandwidth and threshold at CF were analyzed. For multi-frequency stimulus ensembles presented at 65 dB SPL, we evaluated 259 STRFs based on the 2–40 Hz LFP (and 4–8 Hz, 8–16 Hz, and 16–40 Hz filtered versions thereof) and MSUs recorded on the same electrode.

### LFP- and MSU-based frequency-tuning curves

**Figure 2:**
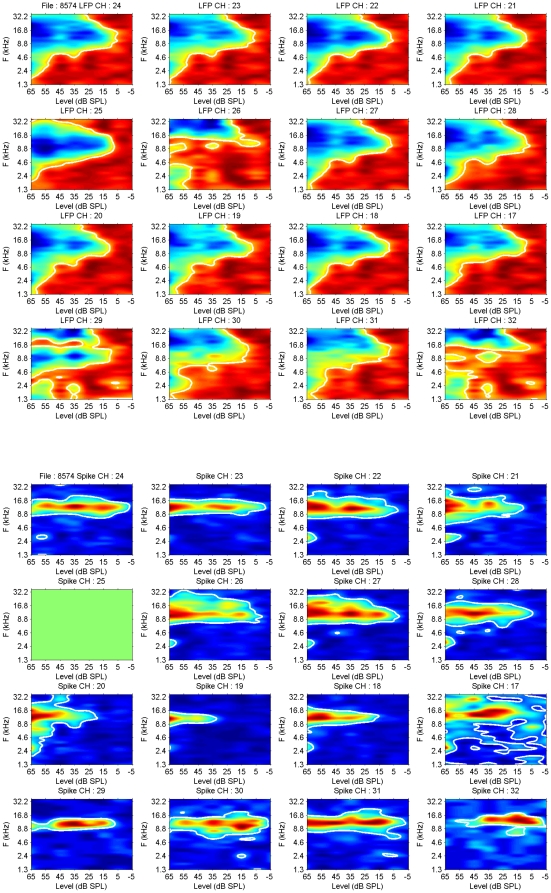
Comparison of frequency-tuning curves (FTCs) for LFP and MSU activity. The top half shows the LFP-based FTCs in color code; blue represents negative values, red positive ones. The 25% of negative peak amplitude contour lines are drawn in (white). The bottom panels show the MSU spike peak firing rates in 5 ms bins indicated in color. The 25% of negative peak amplitude contour lines are drawn in (white). It is noted that the CF of the spike activity corresponds well with that for the LFPs. Spike thresholds are in about half the recordings a few dB more sensitive than the selected LFP level, and in the other half up to 10 dB less sensitive. Channel 25 did not record spike activity.

Figure 2 shows a comparison of 2–40 Hz LFP-based and MSU-based FTCs recorded on the same electrode. The top part of the Figure indicates color-coded LFP peak amplitude (blue is most negative, redish-brown is least negative) with the 30% of negative maximum contour lines (white) drawn in. Each panel is scaled on its own extremes. In the bottom part, the spike peak firing rates in the PSTH (5 ms bins) are indicated in color, and the 30% contour lines are superimposed. Again each panel is scaled on its own extremes. It is noted that the CF of the spike activity corresponds generally with the CF for the LFP contour. Spike thresholds are in about half the recordings a few dB more sensitive than the selected LFP level, and in the other half up to 10 dB less sensitive. The spike-based FTCs are much narrower than those based on LFPs (Table 1). All differences between spikes and LFPs are highly significant. An estimate of the symmetry of the FTC is provided by a comparison of the geometric mean (defined as √(HF*LF) of the high frequency (HF) and low frequency (LF) border of the FTC at 20 dB above CF threshold (geomean bandwidth), and the CF. As can be seen from the Table, these values are very similar, implying FTC symmetry at 20 dB above threshold for both MSU and LFP.

**Table 1:** Comparison of averaged FTC parameters based on LFP and MSU recordings.

Parameter	LFP (SD)	MSU (SD)	N	P-value
CF (kHz)	9.7 (4.7)	11.7 (7.1)	398	<0.0001
Geomean BW (kHz)	9.7 (5.1)	11.5 (6.7)	398	<0.0001
Threshold (dB SPL)	12.3 (9.6)	17.6 (13.4)	398	<0.0001
Bandwidth at 20 dB (oct.)	2.89 (1.18)	1.68 (0.83)	398	<0.0001
CF-change across array (oct.)	0.42 (0.42)	0.96 (0.90)	36	<0.005
CF-change across array (oct.) unpaired	0.46 (0.51)	0.91 (0.88)	148	<0.0005

**Figure 3:**
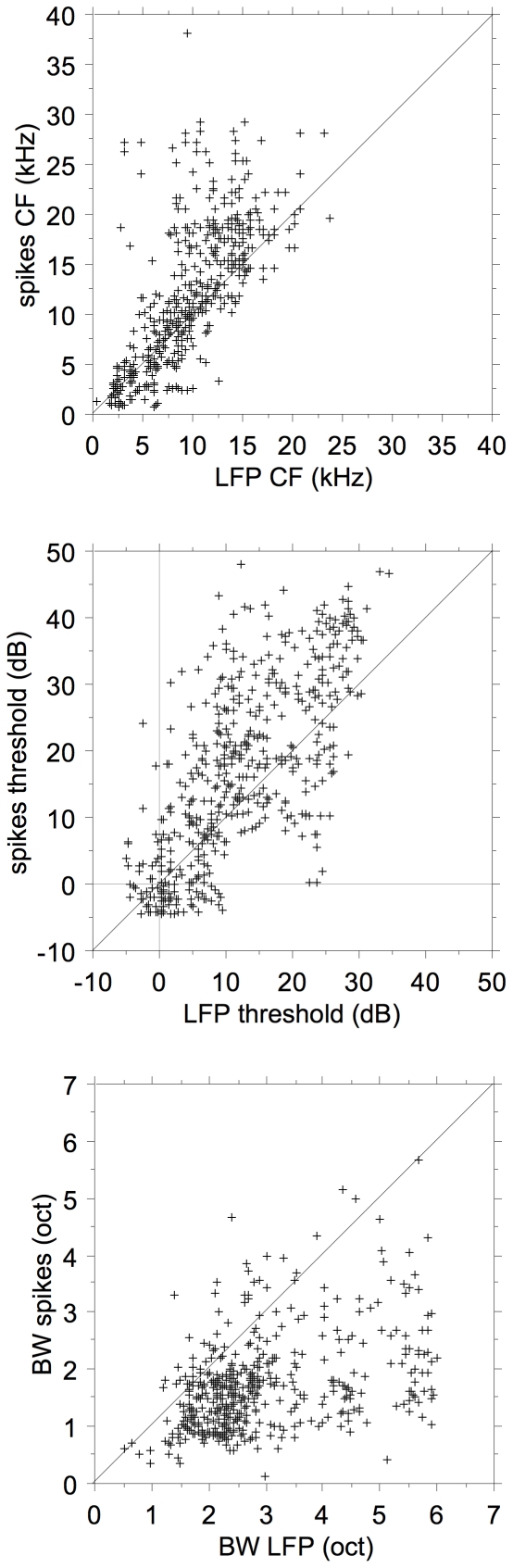
Comparison of LFP and MSU FTC parameters. Top figure compares the CFs for MSU and LFPs, the middle part compares the MSU and LFP thresholds, and the bottom part the FTC bandwidths at 20 dB above threshold.

Pair-wise comparisons of the CFs, the thresholds at CF, and BW at 20 dB above threshold for spikes and LFPs are shown in Figure 3. For the CF one observes a fairly good similarity between LFPs and MSUs below 10 kHz; above that frequency, the CFs for the spikes occupy a large range of values specifically above the LFP CF. It is interesting that there are hardly any CFs>20 kHz for LFPs, whereas for spikes they go routinely to 30 kHz in our recordings. On average, spike thresholds are significantly higher than LFP thresholds, and spike bandwidths are smaller, as summarized in Table 1.

The frequency selectivity of spike and 2–40 Hz LFP data is reflected in the gradient of the CFs across the electrode arrays. Because the distance along the array between the end electrodes is 1.5 mm, one expects a systematic difference between CFs across the array. This difference will be larger when the arrays are aligned along the tonotopic gradient compared to a more oblique orientation. We compared the CF difference for spike-based CFs and LFP-based CFs first in unpaired comparisons (because we have more recordings with LFP-based FTCs at the end electrodes of the array than for spikes). The result shown in the last row of Table 1 suggests a systematic difference along the array with the mean CF difference (in octaves) for spikes being about twice as large as for 2–40 Hz LFPs. The same difference was found for paired comparisons (N being reduced here from 146 to 36) as shown in the last but one row of Table 1. This smaller CF gradient for LFPs is likely related to their FTC BW being a factor 1.7 larger than for the spike-based FTC.

### LFP and MSU based STRFs

**Figure 4:**
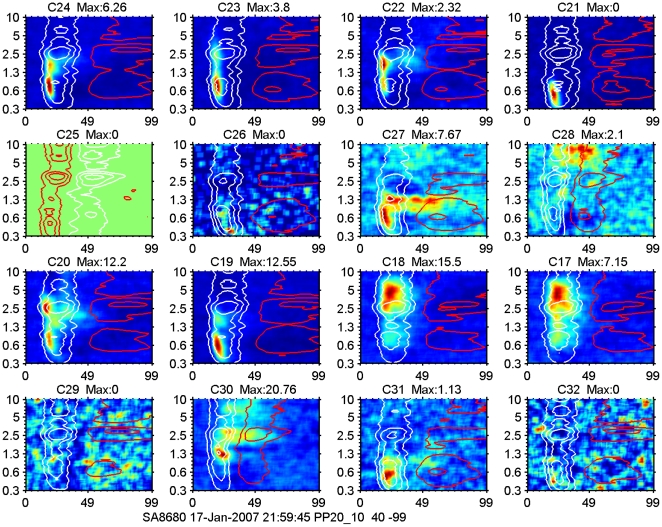
Example of spike-based and LFP-based STRFs for one 16-electrode array recording. Each panel is scaled on its own extremes; red colors indicate high spike firing levels and blue colors low firing levels. Maximum values for the spike responses (number of spikes/bin/stimulus) are indicated above each panel. In some cases this value is 0 whereas there is still some response visible. We use 0 when the value is <0.01 spikes/bin/stimulus. Contour lines for LFP amplitude are white for negative values and red for positive values. The frequency axis (log scale) runs from 300 Hz to 10 kHz and covers 5 octaves. The spatial orientation of the 16 electrodes in this Figure is such that the top two rows are physically on the left of the bottom two rows. Within a row the electrodes are separated by 250 µm, and between rows by 500 µm. Although there is very little difference in the LFPs recorded on the various electrodes in the array, the spike STRFs are varying across the array. For instance the neighboring electrodes 19 and 20 show very different spike-STRFs and nearly identical LFP-STRFs. Here the LFP contours, especially the 25% of peak amplitude, cover about 5 octaves, whereas the neural activity covers 1-2 octaves. Spike firing occurs frequently at the short latency edge of the 50% contour (e.g., top row), but can also be within the 50% contour band (third row, last two columns), or extend beyond the negative amplitude contours (second row, last two columns). Channel 25 did not produce spikes but a clear LFP-based STRF starting with a positive phase and indicative of local hyperpolarization.

An example of spike-based and 2–40 Hz LFP-based STRFs for one 2×8-electrode array recording is shown in Figure 4. Stimulation was at 65 dB SPL with a 20/s multi-frequency stimulus covering the frequency range from 300 Hz to 10 kHz. The spatial orientation of the 16 electrodes in this Figure is such that the top two rows are on the left (and in vivo posterior) of the bottom two rows. Within a row the electrodes are separated by 250 µm, and between rows by 500 µm.

Note that the 2–40 Hz LFP-based STRFs are indicated here by their 25, 50 and 75% contour lines (white for negative amplitudes and red for positive amplitudes), which are overlaid on the spike-based STRFs (maximum firing rate in red, minimum in blue). Although there is very little difference in the 2–40 Hz LFP-based STRFs (except for C25), across the electrodes in the array, the spike-based STRFs are varying across the array. For instance the neighboring electrodes 19 and 20 (left two columns in the third row) show very different spike-STRFs and nearly identical LFP-STRFs. Here the 2–40 Hz LFP contours, especially the outermost, 25% ones, cover about 5 octaves, whereas the spike activity covers 1–2 octaves. Spike firing occurs frequently at the short latency edge of the 50% negative 2–40 Hz LFP-amplitude contour (e.g., top row), but can also be within the 50% contour band (third row, last two columns), or extend beyond the negative amplitude contours (second row, last two columns). Channel 25 did not produce spikes but showed a clear LFP-based STRF starting with a positive phase that could be indicative of local hyperpolarization. It is also possible that electrode C25, which is at the posterior end of the array, was not inserted as deep as the other electrodes because of curvature of the cortex surface. In that case it could potentially be in layer 2 and would be on the top end of the LFP-generating dipole and hence showing an inverted polarity. This would also explain the lack of spike firing.

**Figure 5:**
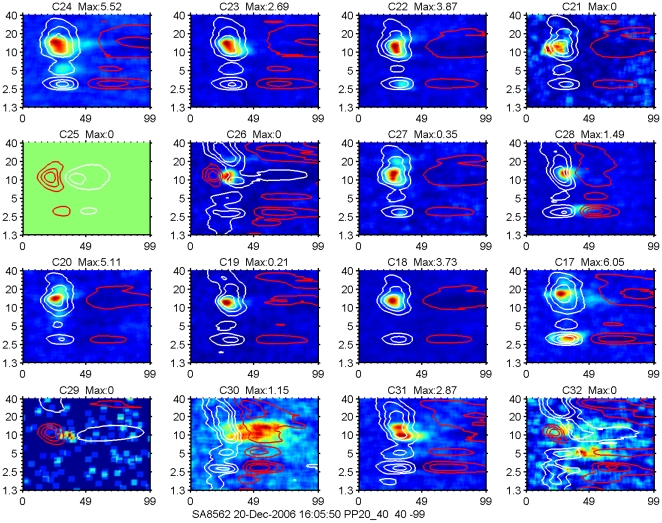
Example of STRFs determined on the basis of spikes and LFPs. STRFs determined on the basis of spikes are shown color coded and LFPs-based ones are indicated by white contour lines for negative levels at 75%, 50% and 25% of maximum, and red ones indicating positive levels. Same plotting conventions as for Figure 4. The frequency range in this example is from 1.2 kHz-40 kHz (5 octaves). As can be seen there are again only minor changes in the LFP profiles across the 8×2 electrode array, whereas the spike-based STRFs are more variable. Note that for a limited frequency range the LFP may start positive, thereby preventing or delaying the spike generation (channels 25 and 26, second row; channels 29 and 32, bottom row).

Another example of STRFs determined on basis of spikes (color coded) and 2–40 Hz LFPs (indicated again by contour lines at 75%, 50% and 25% of negative and positive maxima) recorded in AI is shown in Figure 5. As can be seen, there is now more variation in the multi-peaked LFP profiles across the 8×2 electrode array, especially channels 26, 29 and 32 are very different from the others. Note that for a limited frequency range the LFP may start positive, thereby preventing or delaying the spike generation (channels 25 and 26, second row; channels 29 and 32, bottom row). The spike-based STRFs are again quite variable and ranging from single peaked (channels 18–20) to double peaked (channels 17 and 31) to diffuse (channels 30 and 32).

**Figure 6:**
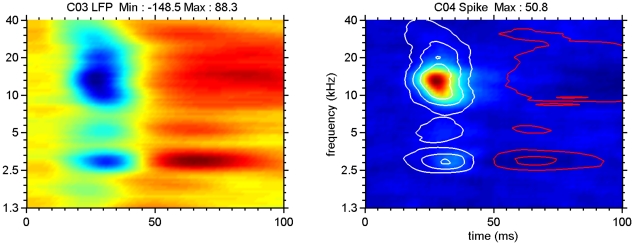
STRFs for LFP (left) and for MSU activity (right) averaged over the 16-electrode array. The two panels represent an average of the data shown in Figure 5. For the left panel blue indicates negative LFP amplitudes, red colors indicate positive amplitude values and yellow corresponds to zero crossings. Contour lines indicate 25, 50 and 75% of LFP negative maximum (white) and positive maximum (red). Note peak spike activity in the 50–75% negative LFP-amplitude contour lines.

The spike- and 2–40 Hz LFP-based STRFs averaged across all electrodes per array for the example of Figure 5 are shown in Figure 6. The left panel shows the color-coded LFP data; negative values are in blue, positive values in red, values close to zero are in yellow. Contour lines in the right panel indicate again 25, 50 and 75% of the LFP maximum (white for negative polarities and red for positive polarities) superimposed on the average spike-based and color-coded STRF. This once more indicates that spike firing occurs preferably near the peak region of the LFP, and is spectrally much more limited compared to the LFP. It also suggests that the summed spike activity across the entire array still represents a more limited frequency range compared to the averaged LFP, which is very similar to that recorded at individual electrodes.

**Figure 7:**
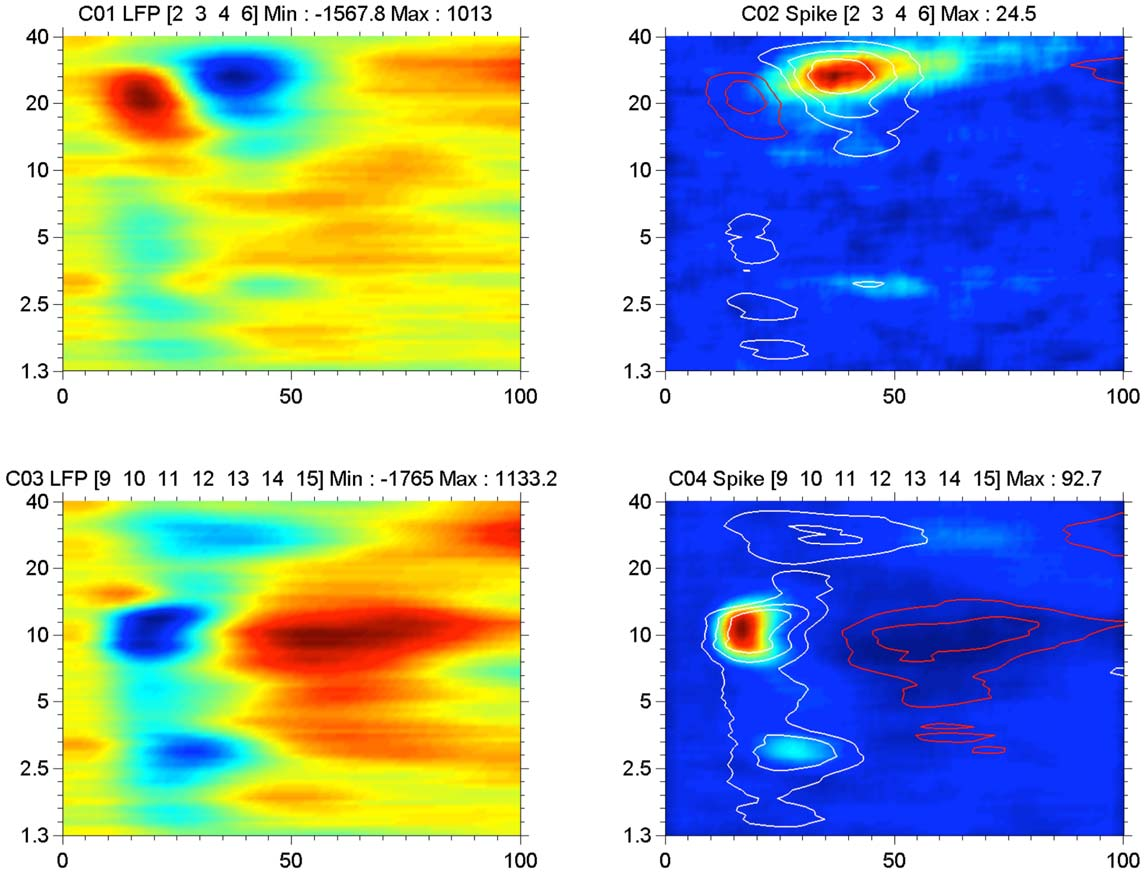
STRFs for LFP (left) and for MSU activity (right) averaged over electrodes in the two arrays that showed clear tuned spike activity. For the top two panels only 4 electrodes, indicated above the panel, produced clear STRFs for spikes. These were averaged. For the top panels array, the LFP-based STRF starts with a positive part followed by a negative part, whereas for the bottom panels (7 electrodes) the common negative-positive sequence is found. The initial positive LFP delays the spike firings for the top array, which occur on the negative going LFP phase. For the bottom array, spike activity occurs for LFP amplitudes that are at least 50% of negative maximum. Although both electrode arrays were in AI and at approximately the same depth, the differences in latencies are pronounced. Contour lines again indicate 25, 50 and 75% of LFP negative maximum (white) and positive maximum (red).

Two other examples of array-averaged STRFs from AI are shown in Figure 7. Here the top row shows an array wherein the 2–40 Hz LFP starts positive for the frequency range of ∼10–30 kHz and the spikes occur at the subsequent negative phase, at longer than usual latencies. For the simultaneously recorded array data shown in the bottom row, the polarity sequence of the 2–40 Hz LFP is the more common negative-positive one. Note again that spikes typically occur within the 50% LFP contour lines.

### Band-pass filtered LFP-based STRFs compared to MSU-based STRFs

**Figure 8:**
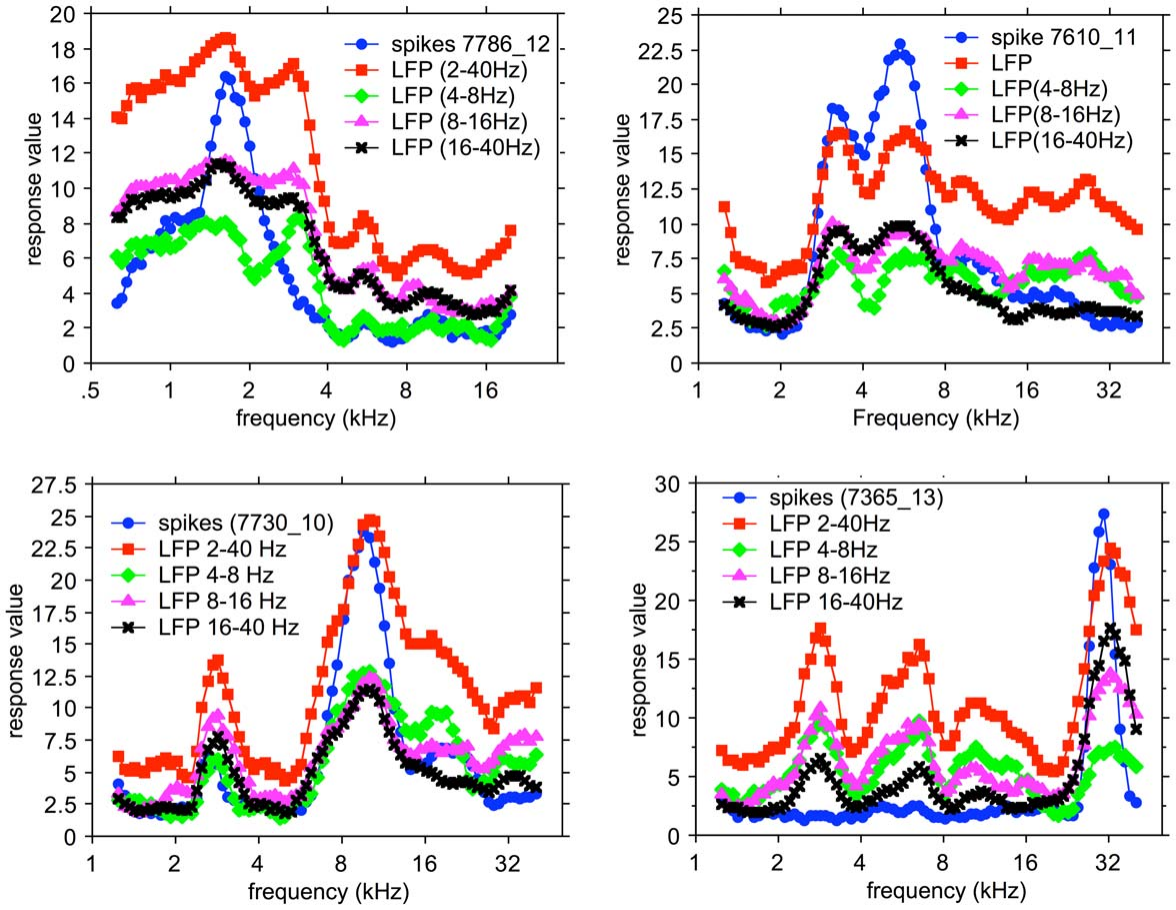
Examples of marginal frequency distributions for STRFs based on 2–40 Hz, 4–8 kHz, 8–16 kHz and 16–40 kHz LFPs, and for spikes for 4 different recordings. In most cases the various filtered LFP data are much broader tuned than the spike based data. A peak in the spike-based frequency distribution always correspond to a peak in the LFP-based distributions. The LFP-based frequency distributions nearly always contain more peaks that the spike-based ones.

Four examples that cover the BF range of the spike based STRFs are shown in Figure 8. Here the marginal frequency distributions of the RMS values (because of the oscillatory character of the narrow-band filtered LFPs) in the 0–100 ms range of the LFP- and spike-based STRFs are presented. We show the amplitudes of the 2–40 Hz LFP (red), the 4–8 Hz filtered LFP (green), the 8–16 Hz filtered LFP (purple), and the 16–40 Hz filtered LFP (black), as well as the peak spike rate in a 5 ms bin (blue) as a function of frequency. The left upper panel shows that the spike activity peaks at slightly below 2 kHz as do the LFP distributions. It is noted that the spike rate distribution is narrow and single peaked, whereas the LFP distributions are all multi-peaked and broader.

The upper right panel shows a double peaked spike-based frequency distribution, which is also present for the LFP-based marginal frequency distribution. Here the Pearson product-moment correlation coefficients between the spike distribution and the 4–8 Hz, 8–16 Hz, and the 16–40 Hz LFP distributions are more variable between the various LFP bands (Table 2). The bottom left panel shows an example where all distributions again are double peaked but with more frequency separation. The bottom right panel shows a unimodal spike-based distribution peaking at 30 kHz but LFP distributions that have additional peaks at ∼3 kHz and ∼6.5 kHz and ∼10 kHz. From the values in Table 2 one notices that for all examples the correlation coefficients are highest between spike-based and the 16–40 Hz LFP band-based distributions. The various band-passed LFP frequency distributions were all highly correlated with the 2–40 Hz LFP: for 4–8 Hz LFP, r = 0.906±0.119; for 8–16 Hz LFP, r = 0.956±0.052; and for 16–40 Hz LFP, r = 0.913±0.071.

**Table 2:** Pearson product-moment correlation coefficients for marginal frequency distributions.

Recording	Spikes vs 4–8 Hz LFP	Spikes vs 8–16 Hz LFP	Spikes vs 16–40 Hz LFP
7786-12	0.754	0.811	0.846
7610-11	0.432	0.743	0.975
7730-10	0.469	0.369	0.565
7365-13	0.205	0.631	0.828
AI (n = 259)	0.512±0.306	0.590±0.266	0.698±0.244

**Figure 9:**
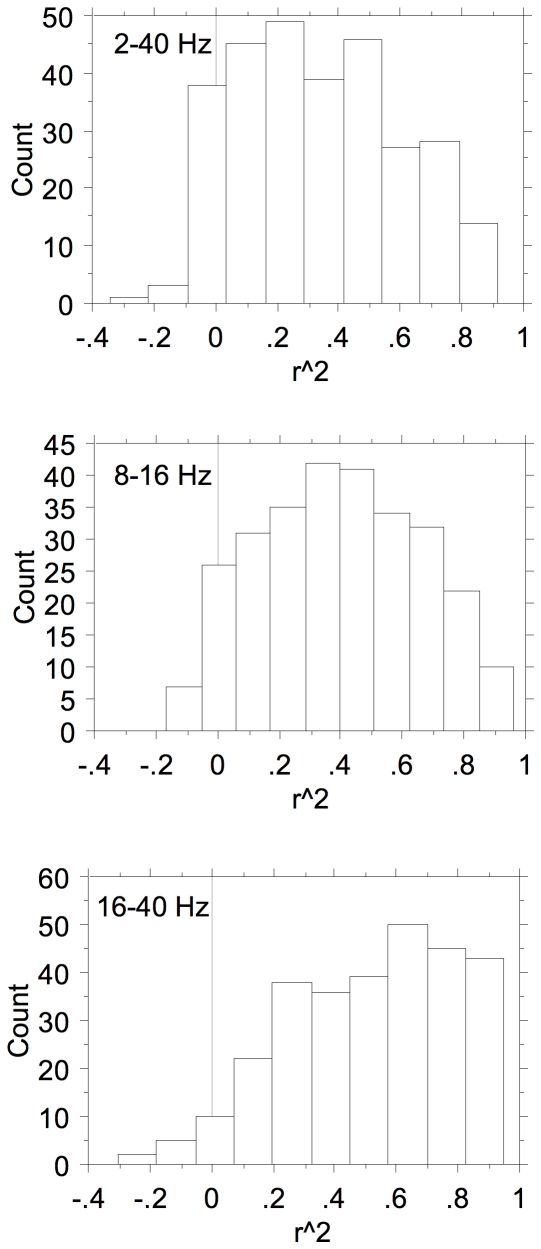
Distributions of the Pearson product-moment squared correlation coefficients. The correlation coefficients are shown (with sign preserved) between the spike-based frequency marginals and the 2–40 Hz, 8–16 Hz, and 16–40 Hz LFP-based frequency marginals.

**Figure 10:**
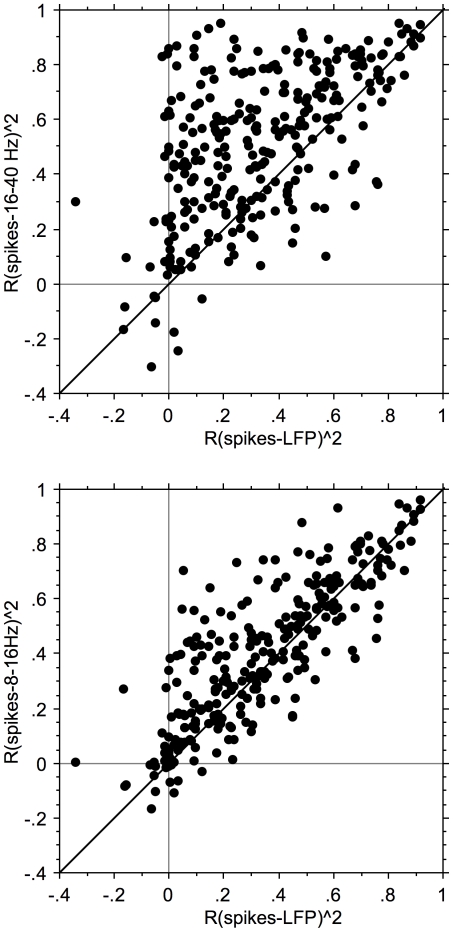
Scattergrams of the Pearson squared product-moment correlation coefficients. The correlation coefficients are shown (with sign preserved) between spike-based and 2–40 Hz LFP-based marginals and spike-based and 16–40 Hz LFP-based marginals (top), and spike-based and 8–16 Hz LFP-based marginals (bottom). The Pearson product-moment correlation coefficients of spikes with 2–40 Hz and 8–16 Hz LFPs are very similar (bottom) whereas those with 16–40 Hz filtered LFPs are clearly different; the Pearson product-moment correlation coefficients with 16–40 Hz LFP frequency-marginals are nearly always larger than those with the 2–40 Hz LFP based frequency-marginals.

The distributions of the squared correlation coefficients between the marginal distributions for filtered LFPs and MSU are shown in Figure 9. The distributions are very broad and are somewhat skewed to lower values for the correlation with the 2–40 Hz LFP and to higher values respectively for the correlation with the 16–40 Hz LFP. This is furthermore illustrated in Figure 10 where the squared correlation coefficients between spikes- (4–20 Hz) LFP against spikes- (16–40 Hz) LFP are plotted (top) and against spikes-(8–16 Hz) LFP (bottom). It is clear that there is a large difference between the correlation of the spike-based frequency distribution and the 2–40 Hz LFP-based one and with the 16–40 Hz filtered LFP-based distribution. The differences for 2–40 Hz LFP and 8–16 Hz LFP (as well as 4–8 Hz LFP–not shown) data are much smaller and suggest that the 16–40 Hz LFPs reflect different processes related to spike generation compared to the <16 Hz LFP data.

### Spontaneous activity cross-correlations

Multi-electrode array recordings of spontaneous activity were made from primary auditory cortex in the same 12 normal hearing cats. We limited the number of spike-spike and (2–40 Hz) LFP-LFP cross-correlations by requiring >200 MSU spikes in 900 s per electrode.

### Comparison of LFP-pair and spike-pair correlation

**Figure 11:**
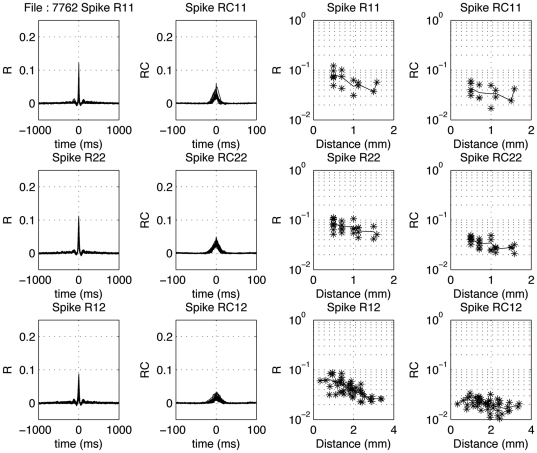
Distance dependence of spike-spike correlograms. In the left column, cross-correlation coefficient functions are show for all pairs from array 1 (top), array 2 (middle) and between electrodes located in different arrays (bottom). The second column shows the coherence-corrected correlograms. Note the extensive overlap of these correlograms. Note that the peak values in the correlogram are smaller between arrays than within arrays. The fourth column upper two rows show the dependence of the corrected peak values as a function of distance for the within-array correlations; one notices only a moderate effect. The mean values are indicated with a thin line. The third column upper two rows show the change in peak cross-correlation coefficient as a function of electrode distance within an array. In the fourth column the corrected peak cross-correlation coefficients for spike-spike pairs are shown.

The first two columns of Figure 11 show uncorrected and coherence corrected correlation-coefficient functions for example spike-pairs recorded simultaneously on a pair of 8-electrode arrays. The cross-correlograms resulting from all pair correlations for the 8 electrodes in an array are superimposed. The top two rows indicate the within-array results, and the bottom row the between-array results. The uncorrected curves typically overlap with little variation. Although small, the spike-spike cross-correlation coefficients were all significantly different from 0 (*P*<0.0001). In the right most two columns the dependence of the peak correlation coefficients on electrode-pair distance is shown. The mean values are drawn in as a thin line.

**Figure 12:**
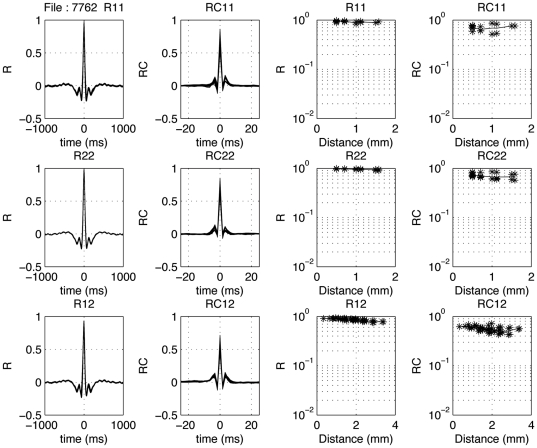
Distance dependence of 2–40 Hz LFP-pair correlograms. In the left column, cross-correlation coefficient functions are show for all pairs from array 1 (top), array 2 (middle) and between electrodes located in different arrays (bottom). The second column shows the coherence-corrected correlograms. Note the extensive overlap of these correlograms. Note that the peak values in the correlogram are similar between arrays than within arrays. The fourth column upper two rows show the dependence of the corrected peak values as a function of distance for the within-array correlations; one notices only a moderate effect. The mean values are indicated with a thin line. The third column upper two rows show the change in peak cross-correlation coefficient as a function of electrode distance within an array. In the fourth column the corrected peak cross-correlation coefficients for spike-spike pairs are shown.

**Figure 13:**
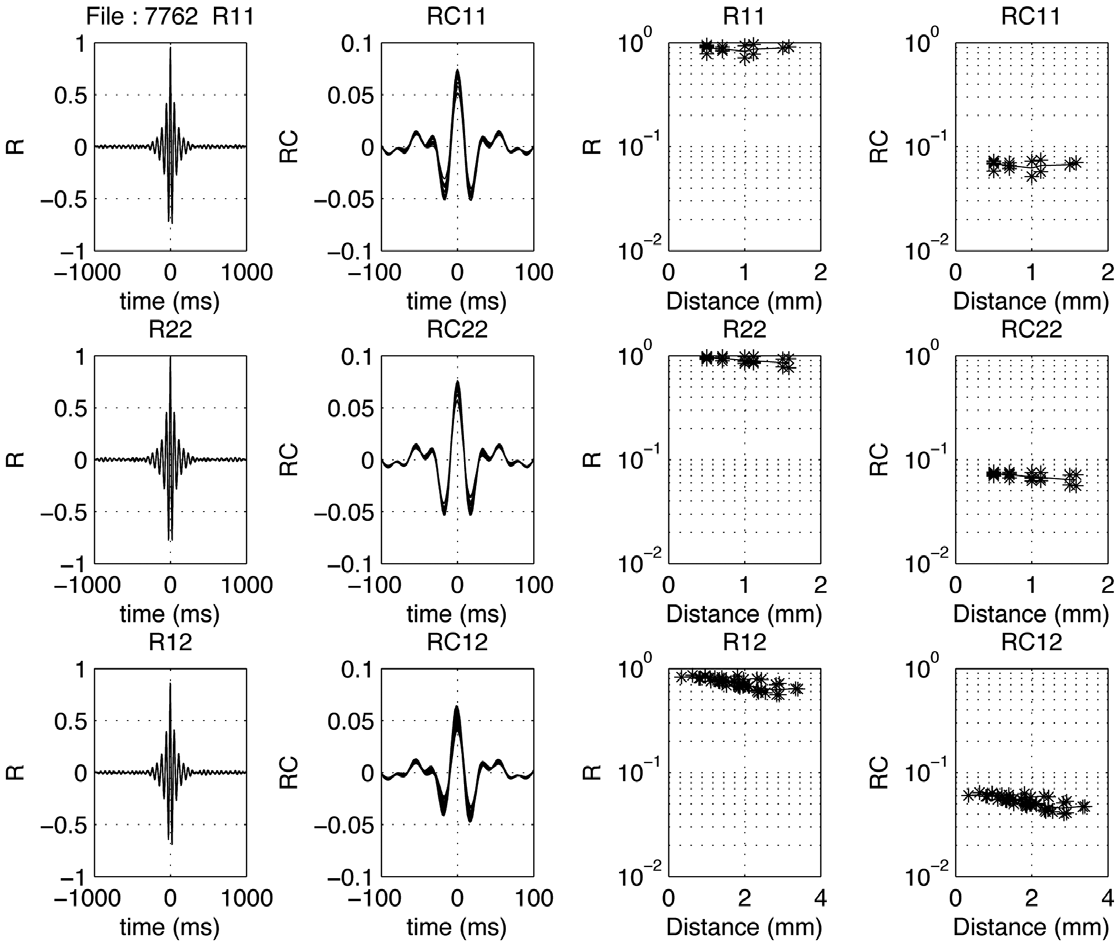
Distance dependence of 16–40 Hz LFP-pair correlograms. In the left column, cross-correlation coefficient functions are show for all pairs from array 1 (top), array 2 (middle) and between electrodes located in different arrays (bottom). The second column shows the coherence-corrected correlograms. Note the extensive overlap of these correlograms. Note that the peak values in the correlogram are similar between arrays than within arrays. The fourth column upper two rows show the dependence of the corrected peak values as a function of distance for the within-array correlations; one notices only a moderate effect. The mean values are indicated with a thin line. The third column upper two rows show the change in peak cross-correlation coefficient as a function of electrode distance within an array. In the fourth column the corrected peak cross-correlation coefficients for spike-spike pairs are shown.

The 2–40 Hz and 16–40 Hz LFP-pair cross-correlation results from the same recording are shown in the same format in Figures 12 and 13. In Figure 13 one observes spindle frequency (∼8 Hz) activity superimposed on the delta activity. Correcting for common periodicities in the cross-correlograms (see Methods) leaves a narrow peak (second column; note the much shorter time scale from −20 to 20 ms or −100 to +100 ms). In the third and fourth columns of Figures 12 and 13 the distance dependence of the peak cross-correlation coefficients within an array is shown for uncorrected and corrected correlograms, respectively. The lines indicate mean values. As shown in the top two rows, there is a systematic small decrease with distance for the LFPs (last column), with the uncorrected cross-correlations (column 3) generally being less dependent on inter-electrode distance compared to the corrected ones.

**Figure 14:**
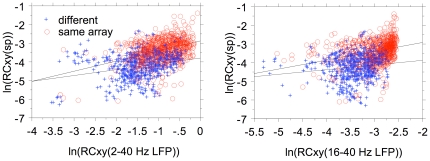
Scattergram of peak correlation coefficients for LFP pairs and spike pairs. Natural logarithm are used for coherence-corrected values. Regression line are drawn separately for within array electrode pairs and for between array electrode pairs.

For 1373 pair correlations we show the relationship (Figure 14) between the natural logarithms of the coherence-corrected peak correlation coefficient values for LFP pairs (horizontal axis) and spike pairs (vertical axis). The dependence is analyzed both for within array (red) and between array pairs (blue). For the relation between the corrected spike-pair correlation coefficients with the 2–40 Hz LFP-pair correlation coefficients the r^2^ values are 0.159 (same array) and 0.076 (different array. For the relation with the 16–40 Hz LFP-pairs the r^2^ values are 0.091 (same array) and 0.023 (different array). Assuming that the LFPs are causal to the spikes, pair-wise correlations between LFPs should be strongly correlated with the pair-wise spike correlations for the same electrodes. Here we only find correlations (r^2^) between about 0.02 and 0.16, suggesting that the unexplained variance is between 0.98 and 0.84. This suggests that the wide band LFP data are predicting more of the variance in the spike-pair cross correlation coefficients than the 16–40 Hz LFP data.

### Distance dependence of LFP- and spike-pair correlations

**Figure 15:**
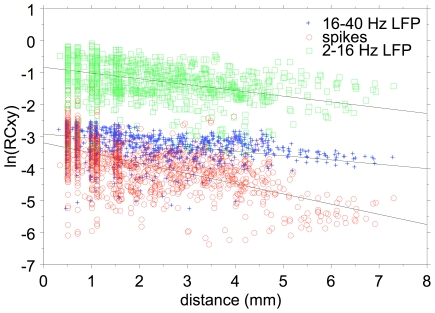
Correlation coefficient distance dependence. Scatterplot of the distance dependence of the natural log of the coherence-corrected peak cross-correlation coefficients for 2–16 Hz LFP-pairs (green symbols), 16–40 Hz LFP-pairs (Blue symbols), and spike-pairs (red symbols) recorded on the same electrodes. The slopes of the regression lines are steeper for the spike data compared to the LFP data.

In Figure 15 we show the dependence of the natural logarithm of the corrected correlation coefficients on electrode distance. The slopes for the 2–40 Hz, 16–40 Hz and spike data are 0.181, 0.136, and 0.317 respectively. This translates in space constants (equal to 1/slope) of 5.52 mm, 7.35 mm, and 3.15 mm respectively. It is noted that the space constant for the 16–40 Hz filtered LFP pairs is about twice as large as that for the MSU pairs. This indicates much less spatial selectivity for the 16–40 Hz filtered LFPs compared to spikes.

## Discussion

We presented comparisons for simultaneously recorded LFP-based and spike-based STRFs and FTCs. These comparisons indicated that LFPs filtered between 2 and 40 Hz are much less stimulus-frequency specific than sorted-spike recordings. The CF gradient over the extent of the array (1.5 mm) for spike-based FTCs was about twice that for 2–40 Hz LFP-based FTCs also indicating a much-reduced LFP frequency selectivity compared to spikes. However, the selectivity improves by comparing marginal frequency-distributions for spikes with those for 16–40 Hz filtered LFPs. This confirms the findings in auditory cortex of awake monkeys [29].

### Effects of anesthesia

The overall effect of pentobarbital on cortical responses to stimulation by pure tones is a decrease in the frequency-tuning curve bandwidth [30]. The bandwidths obtained under free field stimulation in the alert cat [31] were about a factor three larger on average than those reported under pentobarbital anesthesia and closed field stimulation [32]. Free field stimulation under pentobarbital anesthesia also results in a two-fold larger bandwidth [33] compared to closed field stimulation. In the present study, performed under free field stimulation and ketamine anesthesia, the average frequency-tuning curve bandwidth was about twice as large as found by Schreiner and Sutter [32]. This suggests that the difference in cortical frequency tuning between ketamine-anesthetized cats and awake cats under similar stimulus conditions is small. What remains different is that during anesthesia most responses in cat AI are phasic, whereas in awake animals a sizable portion (30%) fires tonically [34].

The STRFs and the frequency-tuning curves in AI are relatively low level characterizations of neuronal filtering resulting from the interaction between excitation and inhibition, which may be different in anesthetized and awake animals. However, comparisons of cortical STRF shapes from ketamine-anesthetized [14] and awake ferrets [35], obtained under passive stimulation conditions, do not point to large anesthesia effects. Passive random chord stimulation in awake monkey AI [36] and ketamine-anesthetized cat AI [15] also resulted in STRFs that were comparable under changes of stimulus density. These are all comparisons based on the shape of the STRF, not on the response strength, which is strongly affected by certain types of anesthesia, especially pentobarbital [30].

Comparing the values of the cross-correlation coefficients for spontaneous spike activity from this and our previous studies [19], [24], [27] in cat AI under ketamine anesthesia with those from Abeles [37], [38], obtained in awake cat AI, suggests that the ketamine anesthesia did not result in major differences. In our data [19] the mean correlation coefficients for single-electrode pairs were, respectively, 0.04 and 0.11 for 1- and 10-ms bin correlograms as compared with Abeles' value of 0.06 for 5-ms bin correlograms. On the basis of the effect of binwidth on the correlation coefficient alone, one would expect for our data a correlation coefficient of 0.08 for 5-ms bin correlograms, which is quite comparable with Abeles' values. The incidence of significant correlations, however, is considerably higher in our ketamine studies compared to Abeles' [37], [38] studies in behaving animals, and slightly higher than in Dickson and Gerstein's [39] cross-correlations from AI in awake, paralyzed animals. Thus, it cannot be excluded that the ketamine anesthesia used in the present study is favorable for inducing correlative behavior between neighboring and distant units. This could have been caused by a ketamine-anesthesia related decrease in uncorrelated, background activity on both neurons relative to the awake condition [40]. The increased background activity in awake animals will reduce the value of the correlation coefficients and in some cases could make them statistically insignificant.

Assuming that the above also hold for LFPs (for which no comparisons as done for spike data could be made), this suggests that the differences observed in the gradient of frequency tuning and space constants for correlated neural activity in the AI of ketamine anesthetized cat are likely representative for what happens in awake cats.

### Short-latency LFP components are causal to spikes

We recorded typically from depths (700–1200 µm) resulting in the shortest spike latencies and initially negative going stimulus-evoked LFPs, and therefore likely corresponding to the thalamo-cortical input layers, lower III and layer IV. The LFPs were obtained by digitally filtering the electrode signal between 2 and 40 Hz, whereas the spikes were obtained by steep filtering between 300 Hz and 3 kHz. It is therefore highly unlikely that spike activity will enter into the 2–40 Hz LFP signal as often suggested [41], [42]. The greater similarity of the LFPs compared to spikes across an electrode array cannot be attributed to a common ground signal (ground electrode was placed in the neck muscles) as the impedance spectrum of cortical neural activity is flat [43]. It is therefore unlikely that the volume conduction of low-frequency LFP activity would be different from that for high-frequency spike activity. It is unlikely that the slow decrease of LFP-pair correlation with electrode distance is due to cross talk between electrodes. The best indication that we are not dealing with crosstalk is that when the electrode array crossed the border between cortical areas there was an abrupt change in LFP amplitude and/or polarity, which is unlikely to happen when there is cross talk between electrodes.

### Frequency-tuning curves

Spike-based FTCs were always contained within the frequency boundaries of the LFP-based ones (Figure 2). Thresholds at CF were comparable for spikes and LFPs but tended to be systematically higher for spikes. CFs were similar below 5 kHz; however, for CFs above 5 kHz the spike-based CFs were systematically higher than the LFP-based ones. The FTC bandwidth at 20 dB above CF threshold was much larger for LFPs compared to spikes: 2.85 oct. and 1.68 oct., respectively. This again is compatible with LFPs reflecting the frequency range of the convergent thalamic input with a wide range of CFs to the cortical cells and the spikes representing the reduced frequency range shaped by intracortical inhibition [10], [11], [12].

The spike-based FTC BW was about twice as large as the average value of 0.8 oct. reported for MSU at 40 dB above the CF threshold by Schreiner and Sutter [32], but very close to the values reported by Carrasco and Lomber [33]. It is likely that the free field stimulus presentation used by us and by Carrasco and Lomber [33] accounts for the difference from the closed field presentation used by Schreiner and Sutter [32].

### STRFs

At a sound level of 65 dB SPL, the STRFs for sorted-spike activity were typically enclosed by the 50% contour lines of the 2–40 Hz LFP-based STRFs. For multiple tuned STRFs (e.g., Figures 5 and 8), spike-based ones were often found covering only a subset of the peaks featured in the LFP-based ones. Even after summing the activities over an entire electrode array the spike-based activity typically covered a much narrower frequency range than the LFP (Figures 6 and 7). The 2–40 Hz LFP-based STRFs were typically similar in shape across a 8 or 16 channel multi-electrode array (cf. Figures 4 and 5) suggesting a generation locus with a radius of >1 mm. This compares in order of magnitude with the results reported in inferior temporal cortex [44], and is at least a factor 4 larger than reported for the visual cortex [45] using an LFP-filter bandwidth comparable to ours. The fact that the spike-based frequency tuning and STRFs were much more confined in the frequency domain also supports the notion that the LFPs, regardless of their filter bandwidth, i.e., the PSPs that they represent, are causal to the spikes rather than the other way around. This was also suggested by our previous study [46] where we modeled spike-based STRFs on the basis of LFP-based STRFs combined with either co-tuned or lateral inhibition.

The STRFs based on 16–40 Hz filtered LFPs were still broader than those for spikes, suggesting that strong inhibition governs the spike-generation process (Figure 8). The shapes of the various LFP-based frequency marginal distributions were generally similar, indicated by correlation coefficients >0.9, suggesting a strong coupling of the various frequency components in the overall wideband LFP signal [47].

### Cross-correlation functions

Cross-correlation functions are dependent on global network properties reflected in oscillations in the delta and sleep-spindle frequency range as well as on local connectivity. One can correct the cross-correlation functions for common periodicities caused by these network oscillations by deconvolution with the autocorrelation functions of the LFPs or spikes [28], [48], which is the equivalent of performing an inverse Fourier transform of the complex coherence function [19]. Here we showed that after such correction (over the frequency range of 0.1–500 Hz), the 2–40 Hz LFP peak cross-correlation coefficients are reduced by at most a factor 2. For spike cross-correlograms the peak width is reduced by a factor 1.5–2.

Rothschild et al. [49], using two-photon calcium imaging in mouse AI, found that the mean signal correlation between all pairs of simultaneously measured neurons was 0.082 (3,926 pairs). This value is higher than the 0.026 for the spike-pairs found in the present study. The difference is likely caused by the pair distances, which were <0.2 mm in the calcium imaging experiments and ≥0.25 mm for the data presented here.

The space constant for the uncorrected LFP-pair correlation coefficients (∼7 mm) was the same as found by Destexhe et al. [18] for LFPs recorded from cat suprasylvian gyrus (Brodman areas 5–8). The large difference in the space constants between spike-pair and LFP-pair correlations is also implied by the poor predictability of MSU spike-pair correlation from the LFP-pair correlations (Figure 14). This again indicates that the LFPs, even after correction for common periodicities resulting from network oscillations, represent a more global measure than the spike activity. However, it also emphasizes that LFPs are not simply volume-conducted activity from within a fixed volume around the electrode tip because in that case the space constants (for these spontaneous activity correlations) would be the same in all cortical areas.

The CF shift across an electrode array for 2–40 Hz LFPs was about 0.46 octave compared to about 0.91 octave for spikes (a factor ∼2 difference). This is comparable with space constant differences of 5.5 and 3.15 mm, respectively, for spontaneous LFP-pair and spike-pair cross-correlations (a factor of 1.75). It is thus likely that even for spontaneous activity, the cross-correlation coefficients still reflect the overlap of the frequency tuning of the neurons [19] for spikes as well as for LFPs.
